# Author Correction: Origin of large plasticity and multiscale effects in iron-based metallic glasses

**DOI:** 10.1038/s41467-018-06284-0

**Published:** 2018-09-11

**Authors:** Baran Sarac, Yurii P. Ivanov, Andrey Chuvilin, Thomas Schöberl, Mihai Stoica, Zaoli Zhang, Jürgen Eckert

**Affiliations:** 1 Erich Schmid Institute of Materials Science, Austrian Academy of Sciences, 8700 Leoben, Austria; 2 School of Natural Sciences, Far Eastern Federal University, Vladivostok 690950, Russia; 3 CIC nanoGUNE Consolider, 20018 San Sebastian, Spain; 4 IKERBASQUE, Basque Foundation for Science, 48013 Bilbao, Spain; 5 Laboratory of Metal Physics and Technology, Department of Materials, ETH Zurich, 8093 Zurich, Switzerland; 6 Politehnica University of Timisoara, 300006 Timisoara, Romania; 7 Department of Materials Physics, Montanuniversität Leoben, 8700 Leoben, Austria

Correction to: *Nature Communications*; 10.1038/s41467-018-03744-5; published online 06 April 2018

The authors became aware of a mistake in the original version of this Article. Specifically, where discussing the Curie temperature of the amorphous phase, *T*_c_, in the ‘Thermal characterization’ section of the Results and in Fig. 2, the authors should have been discussing the Curie temperature of the magnetic crystalline phases *T′*_c_.

As a result of this, the following changes have been made to the originally published version of this Article:

The third sentence of the ‘Thermal characterization’ section of the Results originally incorrectly read ‘Enthalpy of crystallization and melting are measured as Δ*H*_x_ = −108.4 J/g and Δ*H*_m_ = 83.7 J/g, respectively (Fig. 2b).’. The correct version states ‘The Curie temperature of the amorphous phase *T*_c_ is measured as 605 K, while the enthalpy of crystallization and melting are measured as Δ*H*_x_ = −108.4 J/g (Fig. 2a) and Δ*H*_m_ = 83.7 J/g (Fig. 2b), respectively.’

The last sentence of the same paragraph originally incorrectly read ‘A clear curie temperature *T*_c_ is also observed at 945 K (Fig. 2c).’. The correct version states ‘A clear Curie temperature of the magnetic crystalline phases *T′*_c_ is also observed at 945 K (Fig. 2c)’.

The original version of Fig. 2a initially omitted the label ‘*T*_c_ = 605 K’ and the corresponding arrow. In Fig. 2c, the label ‘*T*’_c_ = 945 K’ initially incorrectly read ‘*T*_c_ = 945 K’.

The second sentence of the legend of Fig. 2 originally incorrectly read ‘The glass transition *T*_g_, crystallization temperature *T*_x_, and heat of crystallization Δ*H*_x_ are provided in (**a**).’ The correct version states ‘The glass transition *T*_g_, crystallization temperature *T*_x_, Curie temperature *T*_c_, and heat of crystallization Δ*H*_x_ are provided in (**a**)’.

The fourth sentence of the legend of Fig. 2 originally incorrectly read ‘In (**c**), Curie temperature *T*_c_ of the BMG is shown.’ The correct version states ‘In (**c**), Curie temperature *T’*_c_ of the magnetic crystalline phases formed during the quenching and/or heating step is shown’.

In addition to this, there were errors in some of the equations in the main text, and the glass composition:

The original version of this Article contained an error in the third sentence of the third paragraph of the Introduction, which incorrectly read ‘Besides, it has been reported that a newly developed 1 mm diameter Fe_50_Ni_50_P_13_C_7_ BMG possesses extensive plasticity (up to 22% plasticity) together with a fracture toughness of *K*_C_ ≈ 50 MPa m^−1/2^ with a yield strength of *σ*_y_ = 2250 MPa^5^, where the yield strength and plastic strain with 2 mm diameter samples are 2800 MPa (measured from the deviation point of linearity) and 3%^29^.’. The correct version states ‘Fe_50_Ni_30_P_13_C_7_’ in place of ‘Fe_50_Ni_50_P_13_C_7_’.

The second sentence of the ‘Thermal characterization’ section of the Results originally incorrectly read ‘The extent of the supercooled liquid region (Fig. 2a), Δ*T* = *T*_g_ − *T*_x_, is 24 K confirming the previous findings^5,34^.’. The correct version states ‘Δ*T* = *T*_x_ − *T*_g_’ instead of ‘Δ*T* = *T*_g_ − *T*_x_’.

The second sentence of the first paragraph of the Discussion originally incorrectly read ‘The enthalpy of mixing of Fe−Ni, $\Delta H_{Fe - C}^{mix}$, is very small (−2 kJ/mol) as compared to $\Delta H_{{\rm Fe} - {\rm Ni}}^{{\rm mix}}$ = −39.5 kJ/mol and $\Delta H_{{\rm Fe} - {\rm C}}^{{\rm mix}}$ = −50 kJ∕mol^46^, rendering a low atomic bond force between Fe and Ni atoms.’. The correct version states ‘$\Delta H_{{\rm Fe} - {\rm Ni}}^{{\rm mix}}$, is very small’ instead of ‘$\Delta H_{{\rm Fe} - {\rm C}}^{{\rm mix}}$, is very small’ and ‘$\Delta H_{{\rm Ni} - {\rm C}}^{{\rm mix}}$ = −39.5 kJ/mol’ rather than ‘$\Delta H_{{\rm Fe} - {\rm Ni}}^{{\rm mix}}$ = −39.5 kJ/mol’. The final corrected sentence therefore reads ‘The enthalpy of mixing of Fe−Ni, $\Delta H_{{\rm Fe} - {\rm Ni}}^{{\rm mix}}$, is very small (−2 kJ/mol) as compared to $\Delta H_{{\rm Ni} - {\rm C}}^{{\rm mix}}$ = −39.5 kJ/mol and $\Delta H_{{\rm Fe} - {\rm C}}^{{\rm mix}}$ = −50 kJ∕mol^46^, rendering a low atomic bond force between Fe and Ni atoms.’

The seventh sentence of the same paragraph originally incorrectly read ‘Recent observations for the same composition have postulated that the thermal parameters such as the width of the supercooled liquid region Δ*T*_x_(=*T*_x_ − *T*_g_), the reduced glass transition temperature *T*_rg_(=(*T*_g_)/(*T*_x_)) and the *γ* parameter (=(*T*_x_)/(*T*_g_ + *T*_l_)) defining the glass-forming ability (GFA) of the BMG decrease for more than 5 at.% Ni addition^34^.’ The correct version states ‘*T*_rg_(=(*T*_g_)/(*T*_l_))’ instead of ‘*T*_rg_(=(*T*_g_)/(*T*_x_))’.

The 12th sentence of the same paragraph originally incorrectly read ‘This finding also highlight the fact that the critical casting thickness is only size-dependent and independent of the glassy alloy composition used.’ The correct version states ‘critical casting rate’ instead of ‘critical casting thickness’.

The 14th sentence of the same paragraph originally incorrectly read ‘However, the critical casting thickness for these Fe-based alloy systems can be estimated to be between 10,000 and 20,000 K/s (according to ref. ^50^), which means the nanocrystallization might occur upon quenching of the glassy melt.’ The correct version states ‘critical casting rate’ instead of ‘critical casting thickness’.

This has been corrected in both the PDF and HTML versions of the Article. While the Curie temperature of the glass is lower than previously reported, this error does not affect the original discussion or conclusions of the Article. The authors apologize for the confusion caused by this mistake.

